# Exploring animal husbandry in smallholder dairy systems in Ethiopia using photovoice

**DOI:** 10.1186/s40066-023-00420-w

**Published:** 2023-06-14

**Authors:** Ndungu S. Nyokabi, Lisette Phelan, Gizachew Gemechu, Stefan Berg, Adane Mihret, James L. N. Wood, Henrietta L. Moore

**Affiliations:** 1 Institute for Global Prosperity, University College London, London, UK; 2 School of Geography, University of Leeds, Leeds, UK; 3 Armauer Hansen Research Institute (AHRI), Addis Ababa, Ethiopia; 4 Bernhard Nocht Institute for Tropical Medicine, Hamburg, Germany; 5 Department of Veterinary Medicine, University of Cambridge, Cambridge, UK

**Keywords:** Zoonoses, Animal health, Dairy production, Participatory research, Local knowledge, Documentary photography

## Abstract

This study uses photovoice to explore smallholder dairy farmers’ husbandry knowledge and practices and document how they address constraints faced in pursuing their livelihood strategy. Currently, there is a paucity of farmer-led research in Ethiopia which captures farmers’ local knowledge and lived experiences.

This study was conducted in April and May 2021 in Kaliti, a sub-city of Addis Ababa, and Holeta, located near Addis Ababa, in the Oromia region of Ethiopia. Farmers were selected through purposive and snowball sampling approaches based on their previous participation in a bovine tuberculosis study. Farmers selection was based on their experience in dairy farming and willingness to attend research-related meetings and to engage in photo-taking and subsequent group discussions. Farmers were trained on the use of the digital camera and asked to take pictures of their day-to-day activities, challenges faced in pursuing dairy production and how they overcome these challenges.

The pictures taken by farmers indicated their attachment to their cattle, cattle disease symptoms, manure management, pest control practices, cattle housing, feeding practices, milking hygiene and storage. Discussions revealed that husbandry challenges faced stemmed from land-use change, declining farm sizes, poor access to veterinary and animal health services, low milk prices and high cattle feed prices. Farmers explained that they had developed knowledge of cattle nutrition, such as feed ration mixing and ways to deal with manure problems. The results of this study underscore that farmers have a good understanding of husbandry challenges and, additionally, have a wealth of local knowledge which can be leveraged, if captured through participatory and visual research methods, such as photovoice, by policymakers to develop context-aware policies and interventions and recommendations regarding improved practices which are economically viable, and socially and culturally acceptable.

## Introduction

Animal husbandry is typically explored by researchers at the farm level. However, there is ironically a paucity of research which is farmer-led capturing local knowledge and lived experiences [1]. One means of redressing the lack of farmer-led research is to employ participatory visual research approaches, such as photovoice, which give farmers the opportunity and agency to communicate their own stories [1, 2]. Photovoice, developed by Wang and Burris [3], is a methodology which empowers individuals from traditionally marginalised communities to record and reflect on their strengths and concerns, engage in critical dialogue and generate knowledge about important issues impacting their lives and livelihoods [4, 5]. It is a research approach which involves the discussion of photographs, taken by research participants, in small and/or large group settings to generate data that informs policymaking and ensures the design and implementation of socially aware, context-specific policies [4].

A radical, political, humanistic, emotional and compassionate grassroots research method, photovoice, is rooted in problem-based inquiry [5] and draws on three distinct theoretical frameworks: empowerment education for critical consciousness, feminist theory and documentary photography [4, 6, 5]. It is an action-oriented, participant-directed method premised on the idea that individual and community involvement in research and development processes is key to realising social equity [5].

Photovoice democratises research processes by placing analogue or digital cameras in the hands of research participants, such as smallholder farmers, who are typically silenced in research processes. The use of photography as a medium of data collection enables individuals or groups of people to capture and narrate events and situations encountered in their everyday lives and produce an emotional testimony of their lived experiences which represents that of the broader target research community to which they belong [7, 2]. Photovoice constitutes an important research tool which can generate accurate information and catalyse social change [8, 5].

Smallholder dairy farmers play an important role in the functioning of dairy value chains; and local and global food systems [9, 10]. However, they are typically not involved in the development of policies and interventions aimed at enhancing the sustainability of their approaches to milk production; the safety of milk and other dairy products traded and sold to consumers; and food security at a household and community level. Policies and interventions designed to realise behaviour change among farmers and enhance the sustainability of dairy production strategies and systems, and dairy value chains often fail to increase the uptake of technologies which can facilitate sustainable intensification. This is a consequence of policies and interventions not being aligned with farmers’ social, economic, gender and cultural contexts [11, 12]. For example, agricultural interventions may challenge the social fabric of a farming community, by demanding a change in gender roles or requiring greater labour investment, which leads to failure to adopt technologies and/or dis-adoption of technologies [11]. Policymakers’ failure to see agricultural problems through “farmers’ lenses” leads to a reality gap between policy and development interventions and social-economic outcomes [13, 12].

This study uses Ethiopia as a case study for several reasons. Ethiopia has the largest cattle population in Africa [14], and livestock production plays an important role in its agricultural economy, farmers’ livelihoods and food security [14, 15]. Dairy production, an important subsector of livestock production, has seen rapid growth due to increasing demand for milk and milk products, particularly in urban areas, driven by population growth and improved standards of living [9]. In a bid to increase milk production, the Ethiopian government has made significant investments in the intensification of dairy production systems and the promotion of exotic and cross breeds cattle [9]. Livestock husbandry challenges occur simultaneously in farm-level settings. For example, a smallholder dairy farmer may simultaneously face high feed prices and animal health challenges and receive low milk prices [16]. In such a circumstance, policymakers’ efforts to address a husbandry challenge in isolation through a specific intervention—for example, increasing milk production, reducing disease burdens or improving feeding strategies—may not lead to improved animal husbandry outcomes and, thus, the increased sustainability of a farmer’s livelihood strategy and livelihood security [17–19]. There is a need to co-generate interventions with farmers and learn from their experience regarding what can work or fail given the prevailing farm contextual factors [11, 12, 2].

To the best of our knowledge, the livestock husbandry knowledge and practices of smallholder dairy farmers have not been holistically explored through farmer-led photographic or participatory studies in Ethiopia or elsewhere in sub-Saharan Africa, particularly in the study of zoonoses and cattle husbandry. The objective of this study was, therefore, to (1) use photovoice to document and explore smallholder dairy farmers’ husbandry knowledge and practices in smallholder dairy farms in Ethiopia, (2) identify measures that farmers employ to address animal husbandry constraints faced in pursuing their livelihood strategy, and (3) explore farmers experience about photovoice and its value as a research approach.

## Methodology

### Study area

This research was conducted in April and May 2021 in Kaliti, a sub-city of Addis Ababa, and Holeta located in the surrounding Oromia federal region of Ethiopia. The study area was selected for several reasons. First, the area was part of the Ethiopia Control of Bovine Tuberculosis Strategies (ETHICOBOTS) project, an ongoing study on the control of bovine tuberculosis in dairy systems. Second, the area is important for producing milk traded and consumed in Addis Ababa [14, 15]. Third, due to the rapid urbanisation occurring in Ethiopia, the area was deemed representative of feeding and other challenges faced by smallholder dairy farmers as a result of reduced land availability [20]. Finally, the area was regarded as reflective of milk quality challenges faced by actors participating in the dairy value chains in Ethiopia, where poor milk quality is a public health concern which stems from poor milking and handling hygiene and animal health practices at the farm level [20, 15, 21].

### Recruitment of study participants

The selected farmers were from a group who had previous been engaged in the ETHICOBOTs project. Farmers who participated in this study were selected through purposive and snowball sampling approaches. The inclusion criteria were as follows: (1) a minimum of 3 years of experience in dairy farming, (2) willingness to attend research-related meetings and participate in training related to the use of digital cameras provided by the researchers and (3) willingness to engage in photo-taking and subsequent follow-up group discussions to discuss photographs. We looked for a sample that was representative of the gender makeup of the target population of dairy farmers in the study area, and the socio-economic groups, farm sizes and dairy production systems present in rural and urban and peri-urban locations in the study area. Participating farmers were compensated the equivalent of three days’ work pay for the opportunity costs associated with partaking in the study rather than engaging in dairy production activities.

Informed verbal consent was obtained from the farmers who were briefed in the presence of a witness (local experts) that their participation in this study was voluntary and that confidentiality would be maintained. The research received ethical clearance from University College London’s Research Ethics Committee (UCL-REC), with the ethical clearance approval number being 19867/001, as well as from the Armauer Hansen Research Institute (AHRI) and ALERT hospital AHRI/ALERT Ethics Review Committee (AAERC) approval (Protocol number PO-(46/14).

### Research design and data collection

This study employed a modified photovoice process, as described by Bennett and Dearden [22], which entailed the following six steps. Firstly, research participants were recruited using purposive and snowball sampling, and photovoice training workshops were organised. During this training workshop, the concept of photovoice was explained and each participant received training on the use of the digital cameras which were subsequently provided. The research objectives and scope of this research were also outlined: (1) use photovoice to document husbandry knowledge and practices in smallholder dairy farms, (2) identify measures that farmers employ to address animal husbandry constraints at the farm level and (3) explore farmers experience about photovoice and its value as a research approach.

Secondly, each participating farmer was given one digital camera to take photographs of their day-to-day on-farm activities over a week-long period. In total, the researcher distributed cameras to 30 farmers participating in this study, 15 farmers received cameras in Kaliti and 15 farmers received cameras in Holeta. AGFA DC5500® digital cameras were provided as these were judged to be easy to use and could be used for several days on a single charge. Regular contact was maintained with the farmers via phone to address any technical issues faced in the use of the cameras. After one week, the cameras were collected and the photographs were downloaded for printing. In total, farmers took over 3,345 pictures. The researchers went through all the pictures captured by farmers and selected 500 pictures that were deemed to be clear and of sufficiently good quality for printing, to be used for in-depth semi-structured group discussion sessions.

Thirdly, in-depth, semi-structured discussions were conducted to understand farmers’ motivation for taking each printed photograph; the narrative behind each selected picture was captured using a dictaphone, with farmers asked to explain each photograph, before other farmers were invited to further discuss the photograph in question. The discussions were conducted in the Amharic language in Kaliti sub-city and the Afaan Oromo language in Holeta.

The fourth step of the photovoice research process consisted of an initial analysis of the recorded interviews, which included preliminary coding of farmers’ narratives. The final transcripts of the discussions transcribed verbatim before being translated to English by a trained research assistant with a good command of both local languages were compared with the original recordings and memos compiled by the research team during the discussions to ensure consistency and ensure that concepts were not lost during translation.

Thematic content analysis was undertaken using NVIVO software^®^ and followed the grounded approach process as described by Bennett and Dearden [22, 23]. The first step of the analysis involved reading and rereading the transcripts by the researchers to familiarise themselves with the data, including the initially coded themes. The issues discussed by the photovoice participants were grouped into themes that reflected the farmers’ narratives regarding animal husbandry challenges faced and the innovative ways they overcame these challenges. This step was followed by an in-depth analysis, with emerging themes identified and included as appropriate. Supporting verbatim quotes from the discussions were identified based on their capacity to explain farmers’ views on the various identified themes and important findings.

Due to restrictions on organising large group meetings during the COVID-19 pandemic and the civil war in northern Ethiopia, the fifth step of the photovoice research process, which equates to the production of ‘photo books’ and a community exhibition of photographs and follow-up discussions, has not yet taken place at the time of write-up of this paper. This paper does not present the results of follow-up interviews and discussions conducted in the context of community exhibitions due to the logistical challenges arising as a result of the COVID-19 pandemic. This step will take place at a future date as a part of a process by which findings are disseminated to the smallholder dairy farming community and dairy sector stakeholders in Ethiopia. The data analysed and presented in this paper are, thus, solely based on the photographs and the discussions held with the study participants.

## Results

A number of themes emerged from the pictures and discussions with smallholder dairy farmers. Farmers documented their lived experiences of the animal husbandry challenges they faced and how they looked to address constraints in their daily engagement in dairy production activities. The photographs that farmers took revealed their ingenuity, experience gained through trial and error, knowledge learned from farmer-to-farmer learning, acquired information from external sources and, in some cases, on-farm innovation of standard practices, to overcome challenges faced as a result of feed, information and infrastructure-related resource constraints in their localities.

### Smallholder farmer identity and connectedness to farming

The pictures taken portrayed smallholder farmer farming identity connectedness to farming and farmer–animal attachment. The majority of farmers indicated that dairy production was a continuation of a way of life passed down from previous generations. They regarded dairy production as conferring a cultural identity and as connecting them to their land.

*“In Oromo culture, someone is not even considered a farmer if he doesn’t have any cattle”* Holeta photovoice discussion (May 2021)

Moreover, they explained dairy production connected past and present generations through associated cultural practices, including the production of artisanal products, such as cheeses, and by-product foods, such as *kitfo*, a ‘raw, lean meat dish’, and served as a repository of knowledge regarding food, milk and meat value addition, and also rites and rituals.

The majority of farmers took pictures of themselves and their family members with their cows and explained that their cattle herds were an integral part of their lives. They reported that their affinity to their herds stemmed from the fact that their cows, in return, ‘*took care*’ of them and their households by providing food, fuel and, notably, companionship.

*“I love cattle more than humans [..] I could be there with them all the time because I have to understand their day-to-day problems”* Kaliti photovoice discussion (May 2021)

“[Cows are] *not merely [..] my property rather I feel for their soul. I feel for them [..] I do whatever is needed [..] I consider them as my kids*” Kaliti photovoice discussion (May 2021)

Despite enjoying a way of life passed down through generations, farmers observed that society’s view of dairy and livestock production was changing and, they remarked this could result in future generations not perceiving dairy farming as an interesting and profitable occupation. This change was already evident in urban areas where farmers were perceived as a nuisance due to environmental hygiene and health concerns related to livestock keeping.

*“The community want to use the cows’ milk [to raise their children] but do not want to see their waste”* Kaliti photovoice discussion (May 2021)

The majority of farmers who participated kept exotic breeds, mainly Holstein–Friesian and their crosses with zebu, for milk production. A significant proportion of the farmers also kept indigenous zebu breeds adapted to the local environment, based on their ability to cope with feed shortages and disease outbreaks. Cows constituted a source of income through milk production and a source of wealth creation and storage through reproduction and calving. Interestingly, dairy farmers also kept bulls for breeding and oxen for ploughing their farms for crop production and/or rented out their draught power to other farmers. Perceiving ploughing as a skill requiring preservation in the advent of modern agricultural developments, farmers remarked that bulls provided a cheap substitute for human labour reducing the need for investments in costly mechanisation of agricultural production. Farmers kept other animals including goats, sheep, chickens and guinea fowl. Additionally, they kept pets, including dogs and cats, for security and rodent control, respectively. In Holeta, in the Oromia region, farmers also kept horses alongside their cattle, renting these out to transport people and goods to their immediate localities.

### Challenges faced by smallholder dairy farming in urban and peri-urban areas

There were several challenges that smallholder farmers faced in urban and peri-urban areas related to small land sizes, low access to feeds and grazing areas and lack of farm waste disposal areas.

### Land-use change and challenges of reducing farm size

Generally, there is tension between urban development and farmers engaging in livestock production as their main livelihood activity. Farmers are losing access to land due to increasing land prices and government policies that limit the amount of land an individual has the right to use. Farmers, especially those in urban areas, reported that they felt as though they would be the last generation to engage in dairy production as their chosen livelihood activity would likely decline over time as a consequence of urbanisation.

*“We have problems with our neighbours [ a neighbouring real estate]. They complained about the sound our cattle make & smell as well. Even yesterday they came to us complaining*” Kaliti photovoice discussion (May 2021)

Farmers believed that, due to reduced land sizes, they were unable to expand their production and that production had stagnated; they had reached the maximum carrying capacity of the land in terms of cattle numbers.

*“Currently I have 20 cows. If it had not been for the shortage of space their number might have reached up to 300 [after over 15 years of farming]”* Kaliti photovoice discussion (May 2021)

*“I myself would have more than 60 to 70 cows if it was not for a limited area, [..]we do not have plans to expand our work [production]”* Kaliti photovoice discussion (May 2021)

Farmers reported that reduced farm sizes made it difficult to construct improved cattle housing, especially in urban areas. Farmers took photographs of the structures which they used to house their cattle, which ranged from improved housing units to improvised housing units. Most of the improvised housing units had poor lighting, ventilation and flooring. Farmers explained that due to the small plots of land they owned, it was difficult to build good housing structures in addition to having a family home and compound. They also alluded to the fact that constructing improved housing was expensive and beyond the financial resources of most smallholder farmers.

*“We want to get permission to build in an area we have. We cannot keep their [cows’] hygiene to the current 4-meter standard. If we build their room on standard 3 meters to build a feeding place on the remaining 1 meter, we will not be able to clear their waste and take it out with a cart. They might sleep on their dung and urine. This is one of the reasons that make it hard to keep them clean”* Kaliti photovoice discussion (May 2021)

### Feed challenges

Small land sizes exacerbated the challenge associated with low feed availability, especially in urban and peri-urban areas where farmers had small plots of land with no land left for fodder production. Farmers were forced to buy feeds when prices were low and conserve these feeds within their farms. In urban areas, there was competition for grass between farmers and buyers who used grass for coffee ceremonies, held in restaurants and eateries, and for decorative purposes during national holidays and religious festivities.

Farmers primarily addressed the challenges associated with feeds by stockpiling an amount of feed in stores sufficient to see them through several months of the dry season when feeds were scarce. Farmers were, however, aware that storage could lead to feed contamination and quality deterioration. Farmers purchased feeds, such as hay, teff stover, dairy meal, noug seed (*Guizotia abyssinica*) cake, brewers’ spent grain and other milling by-products. Mixing these feeds, farmers observed they had devised a strategy to ensure that they were in a position to give cows the ‘right’ quantity of feed, namely, by using a plastic jerrycan holding approximately 20–25 L per cow. They asserted that the mixed rations which they prepared were nutritious and met the daily nutritional needs of their cows. Farmers said they had learned to mix feeds and estimate the ‘correct’ quantities based on experimentation and trial and error. Farmers indicated they had a wealth of local knowledge related to cattle nutrition and feeding based on their years of experience and insisted that this knowledge was relevant even if it was not scientifically validated.

*“There are some things that are not known to you by science but we know through experience”* Kaliti photovoice discussion (May 2021)

### Manure management challenges

Manure management constituted a challenge, especially for smallholder dairy farmers in urban areas due to the small farm sizes.

*“[We have] a problem with waste disposal [….] we have to pay 2000 birr (approx. 50 USD) [every] 2 weeks for the [waste disposal] tanker”* Kaliti photovoice discussion (May 2021)

Manure was not widely utilised as an organic fertiliser in rural areas, with farmers instead depending on inorganic fertilisers provided at subsidised prices by the government. Many farmers disposed of manure in streams, roads or open fields, especially in urban areas where there were no farmlands. However, a considerable number of farmers in rural and urban areas transformed cattle manure waste into cow dung briquettes that they used as fuel for cooking and heating. Burning cow dung briquettes was also reported to repel mosquitoes. Some farmers produced biogas that they used for heating and cooking purposes.

**Fig. 1:**
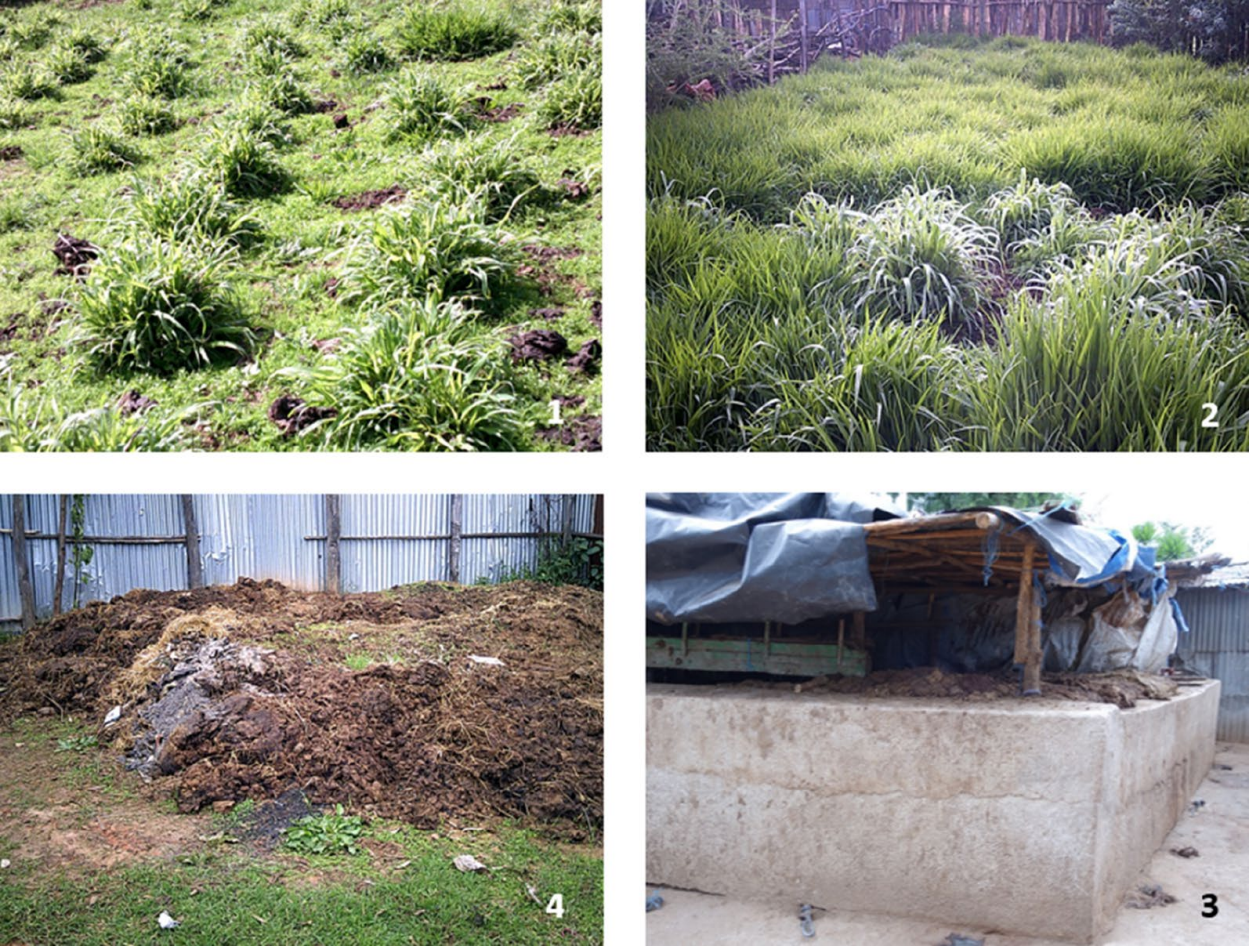
Feed production and manure management (Pictures 1 and 2 Forage grasses, Pictures 3 and 4 Manure storage)

One farmer resident in the urban area with no land in which to dispose of manure had constructed a manure waste pit (resembling a concrete swimming pool structure) (Fig. 1).

Manure from the cattle shed was dumped in the waste pit and emptied when full, roughly once a month. The farmer used a truck to transport the manure from the pit to plant nurseries where seedling growers used the cow dung as a planting medium.

Some farmers with large farms used cow manure as organic fertiliser to produce food and feed on their farms (Fig. 1). One farmer made vermicompost from cow dung which was subsequently used as fertiliser. This farmer explained that making vermicompost helped reduce the smell of the manure, created compost faster and made it easy to manage a large manure volume and the final fertiliser product was of good quality.

### Animal health and welfare

**Fig. 2:**
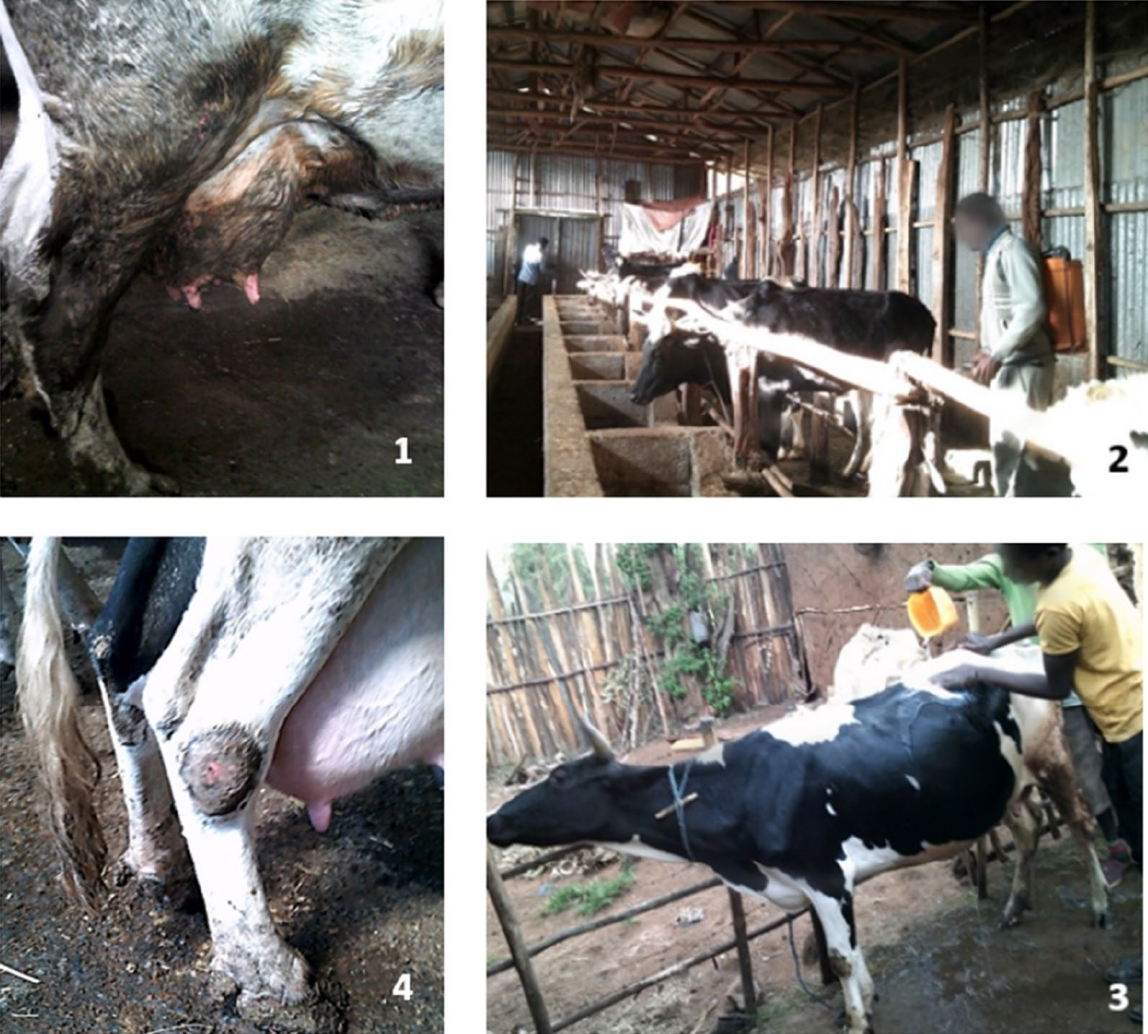
Animal health and welfare practices (Picture 1. Dirty udders, Picture 2. spraying for ectoparasites, Picture 3. cleaning animals, Picture 4. leg wound, washing)

Farmers took pictures of animal disease symptoms which included arthritis, body sores and mastitis. Cattle arthritis and body sores were common in intensive zero-grazing systems where animals were tethered in one spot and floor hygiene was poor, i.e. wet floor and lack of bedding (Fig. 2).

Arthritis and body sores were not captured by farmers who managed extensive systems where animals grazed or had the opportunity to walk outside within the farm compound. Several farmers took pictures of the effects of mastitis on cow teats and udders. Farmers reported that mastitis was a common challenge, especially in intensive zero-grazing systems and explained it could be due to contamination of the teats with bacteria found in cow dung and the floor where the cows slept. One farmer had taken a photo of a cow that had been injured by other cattle stepping on its teats due to the housing unit being overcrowded. A farmer commented:

*“[..]one of the four nipples got swollen and pus with blood was being discharged. After a while, it's [the teat] opening closed, now we are milking her only from three teats”* Kaliti photovoice discussion (May 2021)

Farmers took photographs of themselves cleaning their cows to remove dung from their bodies; they explained this activity was undertaken to maintain cattle hygiene and reduce disturbance by flies (Fig. 2). They also took photographs of themselves spraying their animals to control ectoparasites, a veterinarian helping to treat a cow with a retained placenta, and other veterinarians vaccinating and treating their cows.

*“This is a placenta. I see a difference in their [cow's] milk production when they drop it out by themselves [naturally] and with medical assistance. [Cows’ milk] production decreases when they develop uterine infections that require medical assistance”* Kaliti photovoice discussion (May 2021)

### Milking, equipment and farm hygiene and market challenges

The majority of farmers had established zero-grazing systems which meant cows were milked in the same spot where they slept and consumed feeds. Farmers strived to keep the areas used for milking clean; however, this proved to be a challenge, particularly where a large number of cows were kept in small, confined housing structures. Farmers acknowledged that, at times, milking environments did not meet the required standards to ensure hygienic milking conditions.

*“It is evident that it is not a good practice to milk them [the cows] in their living area [..] [but] we are forced to do that”* Holeta photovoice discussion (May 2021)

Farmers cleaned their cows’ udders and teats with water, or water in combination with soap, before milking to remove dung and other contaminants.

*“We clean them and dry their udder and teats with cloth and milk them at the same spot*” Holeta photovoice discussion (May 2021)

**Fig. 3:**
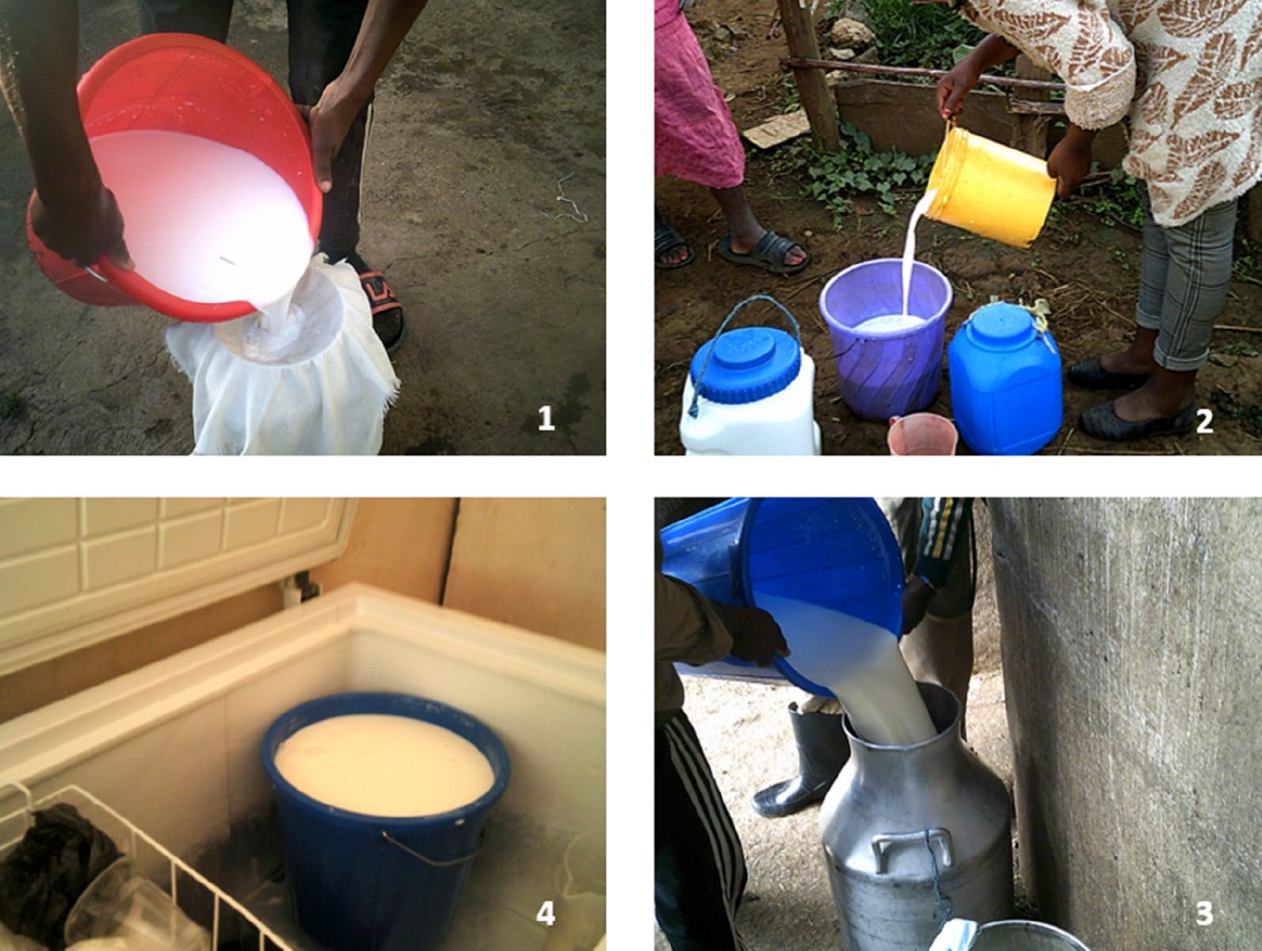
Milk handling practices (Picture 1. Milk sieving, Pictures 2 and 3 milk bulking, Picture 4 milk storage)

Only a minority of farmers used aluminium or steel containers for milking; the majority of farmers used plastic containers despite knowing that this could lead to contamination of stored milk (Fig. 3).

*“Concerning hygiene, we are not able to milk and deliver it in neat materials [aluminium or steel containers] since the cost of such materials are high*” Holeta photovoice discussion (May 2021)

Some farmers sieved milk after milking to remove contaminants, such as cow hair fibres and other particles that they recognised would contaminate the milk leading to spoilage (Fig. 3).

Milk was sold through formal channels which included cooperatives, processors and supermarkets and through informal channels which included small vendors, artisanal butter makers, neighbours and informal traders. Most farmers sold their milk immediately after milking or on the same day. However, some farmers had cooling fridges to store evening milk overnight for sale the next morning. In the formal value chain, at the time of sale, milk was tested using alcohol and density tests. The use of such tests was absent in the informal value chains and the quality of milk purchased was contingent on trust between actors. Milk was bulked by transporters and traders on farms without cooling.

Farmers regarded milk prices as low relative to production and input prices. Milk prices ranged from 18 to 22 birr (around 0.50 US dollars) in rural areas and 25–40 birr (around 0.60–1 US dollars) in urban areas. Farmers complained that similar to informal traders, processors and cooperatives paid low milk prices yet had stricter quality requirements.

“*We purchase 50 kilos of their feed for 800 to 900 Birr (approx. 20-22.50 USD) and sell milk for 18 to 19 birrs (approx.* 0.50 USD)*”* Holeta photovoice discussion (May 2021)

*“We didn’t get any solution from the unions [coops and processors] They buy the same price as merchants [informal traders] do [..] even if we are under the organisations, we are not getting any benefit from it”* Holeta photovoice discussion (May 2021)

### Access to veterinary and animal health services challenges

Farmers stated that access to animal health services was problematic in Ethiopia. Government services, although subsidised, were inefficient and underfunded. Private sector professionals were seen as opportunistic and profit driven rather than intrinsically motivated to serve and meet the needs of farmers.

“*If humans get sick, they go to see a physician. But if animals get sick, it is the physician who has to go and see them. If he does not, they might die. One big problem we have is a shortage of animal health professionals*” Kaliti photovoice discussion (May 2021)

*“There is a lack of animal health professionals who would treat our cows in our home. We are forced to bring them to the [district] clinic to get them treated”* Holeta photovoice discussion (May 2021

Farmers complained of poor animal health and extension services. There was, for example, a shortage of professional inseminators which led to late artificial insemination services and, often, failure. Farmers resorted to using their own or hired bulls as they did not trust that artificial insemination services would be available when their cows were in heat.

*“We do call them while a cow shows those signs for here preparedness; they may not come in the time since they are busy and overloaded [or] they will say there is no vehicle or benzene (fuel)”* Holeta photovoice discussion (May 2021)

*“We are forced to use a bull in our area not to miss the time”* Holeta photovoice discussion (May 2021).

### Participant perceptions on the photovoice approach as a research approach

Farmers were of the opinion that photovoice was an empowering research approach as it provided them with an opportunity to share their knowledge and experience at an individual and collective level with the researcher. They felt that the participatory nature of the process allowed them to directly engage with the researcher in articulating the constraints faced in pursuing their livelihood strategy rather than rely on an appointed representative of their community to do so on their behalf. Farmers expressed confidence that the visual nature of the research method ensured their situation would be understood to a greater extent than if they had been asked to shed light on issues on their behalf through the medium of a key informant interview or focus group discussion. Participants asserted that photovoice and, by extension, the medium of photography enabled them to discuss issues that were either difficult to explain and/or issues that were sensitive that they would normally not discuss with their peers. The photographs they had taken on their farms served as prompts during the follow-up group discussions, reminding participants of issues which they wanted to elaborate on and bring to the researcher’s attention and ensuring that issues that might otherwise have been overlooked or forgotten were discussed. Going beyond that which was visibly captured and immediately obvious to the viewer, participants engaged in critical analysis and provided useful contextual information regarding the photographs that they had taken at an individual level. As the photographs were discussed and issues identified in a group setting, further discussions were triggered, with these discussions providing the opportunity for participants at a group level to ascribe additional meanings to the photographs and determine associated issues which, albeit highlighted by the photographs, may not have initially been apparent even to the photographer.

*“This is research. Educated people shouldn’t do research alone. They have to include the primarily concerned people; cattle owners (main actors) who are responsible for the problems they face as well as share the results gained*” Holeta photovoice discussion (May 2021)

*“I will say the process so far is good. But we have to see it and put it in practice or action. [….] I think the approach is better since we take photos, raise [show] our gaps and weaknesses”* Kaliti photovoice discussion (May 2021)

Additionally, farmers opined that the photovoice activities had allowed them to learn from the experiences of their peers which would enable them to improve their husbandry practices. Photovoice discussion activity allowed farmers to interact and expand their social networks which could enable social learning and could lead to the creation of a community of practice.

*“That is a good thing. For example, if a cow gets sick, rather than telling you the symptoms orally, taking pictures of her appearance, dung everything and showing you, would be preferable. Previously, we had been interviewed a number of times. They had written a lot but we see no use of it”* Holeta photovoice discussion (May 2021)

*“If this meeting was not supported with pictures, everyone would talk only about strengths and we wouldn’t be able to learn from one another […] This has to continue”* Holeta photovoice discussion (May 2021)

## Discussion

This paper employed photovoice “a participant-led photography” to explore smallholder dairy farmers’ husbandry knowledge and practices in smallholder dairy farms in Ethiopia and identify constraints and measures that farmers employ to address them. This study is the first of its kind in that it allowed smallholder dairy farmers who had previously not been represented in research the opportunity to voice their opinions and concerns about livestock production, the challenges and opportunities in Ethiopia. The results revealed that farmers had a wealth of knowledge related to dairy production developed over time through experimentation and trial and error and interaction with other farmers and that they had devised ingenious ways to address the challenges they faced in pursuing their livelihood strategy.

### Smallholder dairy farmers’ husbandry knowledge

The results demonstrated that farmers primarily kept cows for dairy production, and some farmers kept cattle for draught power and/or meat production. Additionally, farmers kept goats, sheep and chicken for cash income, manure, meat and milk. Smallholder farming diversification of dairy production systems through the incorporation of other livestock is in agreement with those of Gebremariam and Belay [24]. Dairy production plays an important role in safeguarding farmers’ livelihood security by providing income through the sale of milk, while herd expansion, specifically, contributes to the creation and storage of wealth. Other livestock serves as an insurance and risk diversification mechanism [24]. However, the presence of other livestock and pets on the farm increases the risk of animal disease transmission. Pets, including dogs and cats, can serve as a reservoir for zoonotic pathogens, e.g. *Salmonella spp*., Q-fever (*Coxiella burnetii*) and cystic echinococcosis (*Echinococcus granulosus*) [25, 26].

Smallholder farmers prefer the indigenous zebu breed adapted to the local environment but with low genetic milk production potential [27]. High milk-producing exotic breeds and their crosses are susceptible to endemic diseases, including bovine tuberculosis and brucellosis, and are not adapted to the hot dry tropical or subtropical climates regions around the world [28, 27, 29].

Our findings prove that dairy production plays an important part in farmers’ everyday lives, not only as a source of income and livelihood but also in conferring a personal and cultural identity and connecting them to their land. Livestock production constitutes a repository of knowledge for the current and future generations, with knowledge passed down from one generation to the next [30, 31]. These findings are in agreement with Garforth [30] who reported that farmers are motivated by a variety of factors to engage in livestock production. These include instrumental factors related to making money and expanding their farm business, but equally ‘social’, ‘expressive’ and ‘intrinsic’ factors related to the prestige and maintaining farming traditions, self-respect and creativity, and the independence and enjoyment of tasks associated with farming [30, 31]. There is therefore a need to support farmers in preserving dairy and livestock production as a way of life and in protecting their cultural heritage amid the ongoing processes of urbanisation and globalisation.

### Smallholder dairy farming husbandry challenges and farmers’ coping measures

Land-use change in Ethiopia is associated with population growth, urbanisation and sub-division of land that has reduced farm sizes and increased land degradation [32]. This land-use change is already constraining dairy production, with farmers no longer in a position to build adequately sized cattle sheds due to decreasing farm sizes, especially in urban and peri-urban areas. Poor cattle housing has animal welfare implications as farmers are forced to zero-graze their cattle, often tethering them to one spot and having reduced movement and hygiene. The continued land-use change will likely force farmers, particularly those with urban and peri-urban dairy systems, to exit and abandon livestock production. This will have livelihood and food security implications not only for smallholder farmers engaged in livestock production but also for the wider population, given the importance of dairy and meat products in ensuring livelihood and food security [32].

It is imperative that there is clear zoning and design of sustainable cities to preserve the ecological, cultural and social values of those engaged in livestock production. This will facilitate the realisation of food security in Ethiopia [33], while not undermining ongoing efforts to establish a circular economy [34, 9].

The results of this study underscore that a decline in farm sizes in Ethiopia has led to a loss of land available for growing feeds and forced farmers to rely on purchased feeds which affect their ability to grow and feed their cattle. This is in agreement with those of Duguma and Janssens [32], who reported that, although Ethiopia has a large dairy cattle population, milk production per cow per day is low. The results of this photovoice study indicate low production could be linked to inefficient nutritional and management practices, the low genetic potential of the indigenous cows, a high prevalence of endemic cattle diseases and parasites, poor access to extension and credit services, and inadequate information to improve animal performance.

Lack of access to quality feeds poses a challenge for dairy farmers in Ethiopia. Purchased feeds exposes farmers to price and feed quality changes. Studies have shown that inadequate quantity and quality of feeds are major limiting factors undermining the development of dairy production in peri-urban and urban areas, with farmers dependent on purchased concentrate and roughage feeds due to limited areas available for grazing [32]. Purchased feeds increase milk production costs and reduce profit margins which adversely impacts farmers’ income and livelihood security [27, 32]. As human populations grow, there is a dilemma on how to use available land, i.e. whether to grow food for human consumption or feed for livestock production [35]. However, as underscored by this paper, livestock production plays an important role in upscaling and converting poor-quality feeds and by-products to quality proteins for human consumption [36].

Manure management constituted a challenge among the respondents engaged in dairy production in urban areas, with manure also not widely used as an organic fertiliser in rural areas as farmers primarily depended on inorganic fertiliser (Fig. 1). This highlights the importance of developing circular, closed production systems that utilise all the resources produced on-farm, including manure and other by-products of dairy production [37]. Manure contains microorganisms, protozoa and viruses, and its management is key to reducing the risks posed to human and animal health [38]. In Ethiopia, the extension system relies heavily on government extension workers who do not typically encourage manure utilisation as their performance is partly evaluated based on the amount of synthetic fertiliser they distribute to farmers [37]. The government has been encouraging farmers to adopt biogas as an alternative to the traditional use of biomass in an open-burning, three-stone stove system [34]. Another potential strategy to utilise manure might be to develop a manure value chain where dairy farmers can sell manure as organic fertiliser to farmers focused on crop production [37].

Farmers reported that they faced pressure from diseases, such as bovine tuberculosis, milk fever, mastitis, and arthritis, and that these diseases were a major constraint to dairy production as they led to economic losses associated with sickness, treatment costs, cattle mortality and morbidity (Fig. 2). These results are in agreement with previous research that reports a high prevalence of endemic animal diseases in Ethiopia, including brucellosis and bovine tuberculosis [20, 39]. Diseases have important public health implications; zoonotic diseases can be transmitted to the general population through the handling and consumption of contaminated dairy products [39]. Farmers face occupational risks associated with exposure to zoonoses due to their close contact with cattle [40, 39]. The results of this paper show that farmers tried to control ectoparasites through spraying and engaged animal health workers to help with disease management. This underscores that there should be a focus on disease prevention on farms rather than the current focus on treatment. Biosecurity adoption will reduce disease pressure, reduce dependence on antimicrobials for treatment, increase food safety and increase farm productivity which will boost farmers’ incomes and livelihoods [25, 26].

The results of this study indicate that veterinary services in Ethiopia are currently underfunded and cannot properly execute their mandate of serving farmers. These findings are in agreement with [20] who have also reported that animal health management in Ethiopia is underfunded which constrains the provision of quality services. It is imperative that the institutional and technological constraints faced by farmers are acknowledged. Currently, government extension services are inefficient and constitute an inadequate level of support for farmers to realise improved dairy production [37]. Low access to capital and information, for example, on animal health and disease control, undermines the development of the dairy sector in Ethiopia [41].

Farmers included in this study implemented milk handling practices which were not in line with the required food safety standards; this could expose milk to contamination, including unclean floors, lack of teat cleaning and the use of non-food-grade plastic during milking and to store milk (Fig. 3). Moreover, milk was transported unrefrigerated which could facilitate bacteria growth and quality deterioration due to the conducive tropical temperatures of Ethiopia [42, 43]. Dirty floors and bedding in cattle sheds and a lack of teat disinfection pose a risk to cattle health and can facilitate the transmission of microbial pathogens which cause mastitis between cows in a herd [42, 43]. Mastitis is a common problem in zero-grazing systems due to poor housing hygiene compared to open-grazing extensive systems [42, 43]. However, some farmers had adopted improved milk handling and hygiene practices, i.e. the use of recommended aluminium and stainless containers, sieving milk, refrigeration of evening milk and alcohol testing by buyers. These recommended containers are of food-grade material and are hygienic and easy to clean and disinfect compared to repurposed plastic containers [44]. Despite farmers and other dairy value chain actors knowing that plastic containers are a risk of milk contamination, they prefer these containers for milk handling as they are cheaper to purchase than aluminium cans [44]. Poor milk quality handling leads to milk contamination and public health risks, including zoonoses [9]. In Ethiopia, the adoption of improved technologies and practices is hindered by low access to credit and information and extension [41]. Improving animal health and hygienic milking and handling could, however, address the current problem of poor milk quality in dairy value chains; this has been also suggested by [45].

The results of this paper reveal that farmers understood the challenges related to dairy production, which is in agreement with previous research conducted in Ethiopia by [21, 40, 46]. Farmers have developed a wealth of local knowledge regarding dairy production through their lived experiences, everyday observations, trial and error experiments, copying and modifying established practices and interventions, and information obtained from their social networks. Similar local knowledge has also been observed in Tanzania by van der Meer et al., [2]. However, this local knowledge is often overlooked by mainstream researchers and policymakers who tend to favour top-down, technocentric interventions and the transfer of technologies without consulting farmers to understand their needs and the prevailing socio-economic context. The results of this study show that farmers have developed a wealth of knowledge and figured out new ways to overcome or mitigate dairy production constraints, such as feed ration mixing, improved feeding and feed conservation. This finding is significant as it agrees with the seminal works of Hill [47, 48] and [49] who asserted that farmers are entrepreneurial and business minded and that their decisions reflect clear and logical thought processes. Moreover, this finding indicates there is a need to prioritise farmers’ perspectives in research processes and recognise that local knowledge has a role to play in generating acceptable solutions to problems facing farmers [47, 49]. It is imperative that policymaking in Ethiopia acknowledges farmers' local knowledge and that interventions are developed based on a consultative and participatory approach that listens to farmers and supports them in addressing challenges faced, given the importance of the dairy sector for food and livelihood security.

We reported in this study that farmers are dealing with multiple challenges simultaneously. Previous studies have shown that dairy farmers face a myriad of problems which require holistic solutions that address the multiple challenges [50]. Piecemeal interventions cannot address farmers’ problems, and thus, it is important to involve them on which important issues should be prioritised rather than impose solutions on them [11, 12]. In Ethiopia and, more broadly, sub-Saharan Africa, the recipients of policy and interventions are typically not involved in the design or implementation of interventions and in agricultural projects, which leads to failure or low sustainability in the long run as the contextual factors shaping the behaviour of smallholder farmers are overlooked [11, 12, 50].

### Photovoice approach: a viable research approach to explore smallholder dairy farming challenges?

Photovoice generates empirical results that are credible and can be accepted by all stakeholders engaged in a research process, given that it constitutes a collaborative and participatory research approach. Photovoice provided the researchers with an opportunity to fully understand the complexities of diseases and the adopted biosecurity measures at the household and community level that other research methods, such as surveys and interviews, may not fully capture. For example, photovoice was able to capture the social impacts of cattle diseases on households and the community. Photovoice can be a tool to facilitate behavioural change by creating awareness and triggering immediate (re)action and planned actions [51]. Cattle disease prevention and the adoption of biosecurity measures can only be successful through strategies that foster collective actions beyond the individual or household level.

The photographs enabled farmers and researchers to visualise livestock production challenges, cattle diseases and prevention measures and facilitated critical face-to-face dialogue and reflection on the issues. The visual element of photovoice invites participants to actively engage with the photographs, and the discussions offer an opportunity to think critically as a group [52]. Photovoice can generate instant behavioural messages through contextually relevant photos and is participant driven [51, 52]. For example, participants became aware of the cattle and human health impacts of their everyday practices and started to think about ways of addressing them.

The findings of this study demonstrate that photovoice allowed access to multiple levels of implicit and explicit knowledge at individual and community levels. Our findings demonstrate how participatory collection of data through a visual research method, such as photovoice, can support the identification of organic, local solutions to problems faced by dairy farmers in Ethiopia. The data demonstrate that there is a considerable scope to tap into farmers’ local knowledge in the context of improving the dairy sector; farmers have ideas as regards manure management in small spaces and improved feeding techniques. Local solutions if scaled up are more likely to be adopted by dairy farmers as they are economically feasible, culturally acceptable and context appropriate [53].

In this study, farmers described their experience of contributing to the research process through photovoice as empowering as they were provided with a platform to communicate their stories in their own words and contribute to the identification of both challenges and potential solutions. Based on the wealth of local knowledge and experience, farmers can be agents for change in their communities [22]. The results of this study suggest photovoice can be a useful first step in a research process as it equates to taking a holistic, participatory and gender-sensitive approach to research and can secure the involvement of farmers in the research process and the conceptualisation, design and implementation of policy and interventions in Ethiopia where there are typically gaps in linking policy and interventions to social outcomes.

In addition to empowering farmers, the process by which photographs were taken and discussed enabled farmers to build new social networks and learn from better-performing peers; the use of photovoice in the research process generated an additional benefit by directly contributing to the dissemination of information and technologies. Mannay [54] opines that photovoice as a research approach enriches a research process by producing data which is directed, constructed and created away from the direct influence of the researcher. It is important to acknowledge the inherent power imbalance which exists between a researcher and the researched as this imbalance impacts the reliability of the data collected [54]. Photovoice directly challenges this power imbalance by providing a platform for the subjects of a research process to tell their stories with the explicit objective being to incorporate their voices into policymaking and the development of behaviour change interventions.

### Methodological limitations

The challenges faced by the researchers while undertaking photovoice research are similar to those highlighted by Bennett and Dearden [22], namely, participant retention, farmers perceiving photovoice as a laborious and tedious process and seasonality, i.e. weather conditions undermining travel on poor road networks. Moreover, one limitation of using photovoice was that farmers could only photograph, and thus capture and communicate, issues that were tangible and observable. Ronzi et al. [52] assert that participants' perceptions are not confined to the photographs that they take. There are opportunities for exploration of the ‘missing photographs’ through other methods such as interviews and focus group discussions to access a broader and deeper level of knowledge and to gain a good understanding of the topic from the participant's point of view.

As researchers, we are reflexive and aware that factors, such as class, age and gender, may have influenced which pictures were taken. This has also been suggested by Bennett and Dearden [22] and Migliorini and Rania [7]. Photographs discussions held to obtain feedback on the use of photovoice as a research method were important in both discussing the intangible and discerning views through an intersectional lens, as advocated by Wang [55] to ensure the generation of policy-relevant findings.

### Implications for policy practice and research

Although this study had only 30 farmers, the photovoice process has the potential to inspire participants to become advocates within their households and the community in general, thus facilitating the diffusion of the issues discussed. Photovoice is intent on grounding knowledge making in community realities, needs and expertise [22, 56]. Photovoice is concerned with reconnecting science with society for social transformation where action and research converge to inform theory in ways that effectively support community advocacy for change [56]. We propose that photovoice be considered and used as a tool by policymakers, researchers and development practitioners on a more routine basis, as a way to involve smallholder farmers in identifying priorities for action and ensuring that their views are included in decision-making and planning processes. Photovoice provides extensive and ‘rich’ data and is well suited to bringing farmers and stakeholders together. Photovoice can enable farmers to articulate the ‘*hidden things’* that are important to people, which researchers or policymakers may not be able ‘to see’ solely through interviews or focus groups [52, 57].

## Conclusion

Dairy farmers in Ethiopia face a myriad of simultaneously occurring problems, including animal health, feed, hygiene, waste management and housing challenges that constrain the development of the dairy sector in Ethiopia. This study highlights the need for holistic policies and behavioural change interventions to be developed and implemented, which acknowledge farmers’ multiple challenges rather than those which prioritise and/or address a given challenge in isolation. Capturing and building on farmers’ lived experiences and engaging them to understand their motivations in addressing challenges faced and opportunities perceived to improve dairy production are key to increasing the likelihood that policy responses and interventions are context aware and appropriate. This will ensure that policy and interventions lead to sustained milk quality improvements and dairy sector growth and development in Ethiopia. Researchers should promote participatory and collaborative learning between farmers and policymakers to reduce power asymmetries and enhance the agency of all stakeholders in processes aimed at enhancing the sustainability of the dairy sector. We conclude that photovoice is a good tool for preliminary scoping and prioritisation of issues and identification of potential entry points for intervention to address these issues based on local needs. Photovoice democratises research processes and beyond giving farmers a voice and can initiate dialogue between dairy sector stakeholders. This is critical to ensuring the acceptability and choice of interventions reflect an understanding and willingness to respond to the concerns of those often marginalised in discourse, debate and policymaking. Photovoice can also be explored in other contexts related to livestock production with larger groups to compare and add additional perspectives.

## Data Availability

The datasets generated and analysed during this study are not publicly available because of privacy concerns. Participants are potentially identifiable due to the small sample size and the qualitative nature of much of the data. The datasets used and/or analysed during the current study are potentially available from the corresponding author on reasonable request.
